# Cancer stem cells from a rare form of glioblastoma multiforme involving the neurogenic ventricular wall

**DOI:** 10.1186/1475-2867-12-41

**Published:** 2012-09-20

**Authors:** Shengwen Calvin Li, Long T Vu, Hector W Ho, Hong Zhen Yin, Vic Keschrumrus, Qiang Lu, Jun Wang, Heying Zhang, Zhiwei Ma, Alexander Stover, John H Weiss, Philip H Schwartz, William G Loudon

**Affiliations:** 1 Neuro-Oncology Research Laboratory, Center for Neuroscience and Stem Cell Research, Children's Hospital of Orange County (CHOC) Research Institute, 455 South Main Street, Orange, CA 92868, USA; 2 Department of Neurological Surgery, Saint Joseph Hospital, Orange, CA, 92868, USA; 3 Department of Neurological Surgery, University of California Irvine, Orange, CA, 92862, USA; 4 Department of Neurology, University of California Irvine, Orange, CA 92862, USA; 5 Department of Biological Science, California State University, Fullerton, CA, 92834, USA; 6 Department of Pathology and Laboratory Medicine, Good Samaritan Hospital Medical Center, 1000 Montauk Highway, West Islip, NY, 11795, USA; 7 Department of Neurosciences, Beckman Research Institute of the City of Hope, Duarte, CA, 91010, USA; 8 National Human Neural Stem Cell Resource, Center for Neuroscience and Stem Cell Research, CHOC Children's Hospital Research Institute, 455 South Main Street, Orange, CA, 92868, USA; 9 Developmental Biology Center, University of California Irvine, Irvine, CA, 92612, USA

**Keywords:** Glioblastoma multiforme, Primary tumors, Brain tumor stem cells, Cancer stem cells, Organotypic brain slice culture

## Abstract

**Background:**

The cancer stem cell (CSC) hypothesis posits that deregulated neural stem cells (NSCs) form the basis of brain tumors such as glioblastoma multiforme (GBM). GBM, however, usually forms in the cerebral white matter while normal NSCs reside in subventricular and hippocampal regions. We attempted to characterize CSCs from a rare form of glioblastoma multiforme involving the neurogenic ventricular wall.

**Methods:**

We described isolating CSCs from a GBM involving the lateral ventricles and characterized these cells with in vitro molecular biomarker profiling, cellular behavior, ex vivo and in vivo techniques.

**Results:**

The patient’s MRI revealed a heterogeneous mass with associated edema, involving the left subventricular zone. Histological examination of the tumor established it as being a high-grade glial neoplasm, characterized by polygonal and fusiform cells with marked nuclear atypia, amphophilic cytoplasm, prominent nucleoli, frequent mitotic figures, irregular zones of necrosis and vascular hyperplasia. Recurrence of the tumor occurred shortly after the surgical resection. CD133-positive cells, isolated from the tumor, expressed stem cell markers including nestin, CD133, Ki67, Sox2, EFNB1, EFNB2, EFNB3, Cav-1, Musashi, Nucleostemin, Notch 2, Notch 4, and Pax6. Biomarkers expressed in differentiated cells included Cathepsin L, Cathepsin B, Mucin18, Mucin24, c-Myc, NSE, and TIMP1. Expression of unique cancer-related transcripts in these CD133-positive cells, such as caveolin-1 and −2, do not appear to have been previously reported in the literature. Ex vivo organotypic brain slice co-culture showed that the CD133+ cells behaved like tumor cells. The CD133-positive cells also induced tumor formation when they were stereotactically transplanted into the brains of the immune-deficient NOD/SCID mice.

**Conclusions:**

This brain tumor involving the neurogenic lateral ventricular wall was comprised of tumor-forming, CD133-positive cancer stem cells, which are likely the driving force for the rapid recurrence of the tumor in the patient.

## Background

Despite aggressive surgery, radiation therapy, and advances in chemotherapy, malignant brain and spinal cord tumors remain a leading cause of morbidity and mortality for children and adults [1,2]. There are few effective treatment options for brain cancer patients, especially for those with diffuse malignant gliomas. The prognosis for malignant brain tumors remains dismal, the long-term survival statistics being very poor. There is also a growing body of data which identify permanent disability among the “fortunate” survivors [3,4]. A fundamentally new research direction to develop new approaches to treat brain tumors is desperately needed.

Cancer stem cells (CSCs) have been defined as immortal cells within a tumor that are capable of unlimited self-renewal and which drive tumor genesis [5,6]. This new insight into the nature of cancer has resulted from the isolation and preliminary characterization of CSCs from many malignancies, including leukemia, multiple myeloma, squamous cell cancer, malignant melanoma, breast cancer, and brain tumors, such as medulloblastoma, ependymoma and malignant glioma [7,8]. Although questioned because of inconsistent biomarker expression [9] and the different purification methods employed [10-12], the CSC model has important implications for cancer therapy.

Normal neural stem cells (NSCs) that have been engineered for tumoricidal activity have been proposed as a novel therapy for malignant brain tumors because they can seek out the tumor cells [13-15]. This is particularly important because diffused glial tumors, brain stem tumors and metastatic tumors may be surgically inaccessible due to tumor growth dispersed throughout eloquent tissues. However, the clinical benefits versus possible detrimental effects have not yet fully been determined. Indeed, normal NSCs reside in the subventricular zone; previous reports have suggested that the tumors involving the subventricular zone of the lateral ventricle might originate from neural stem cells located in the subventricular zone [16-23]. It is well established that the tumor microenvironment plays a critical role for tumor progression. Although they may migrate into the subventricular zone, and hijack and recruit normal NSCs to facilitate tumor progression, malignant gliomas such as glioblastoma multiforme (GBM) usually form in the cerebral white matter.

We have shown that normal stem cells and cancer cells share p53 signaling pathways [24], implying the convergence of stem cells and cancer for signaling pathways [25]. These results prompted us to hypothesize that the convergence of stem cells and cancer may drive tumor recurrence by subclonal switchboard signal activation [26]. Previous reports have presented either a clinical description or molecular and cellular characterization of brain tumors, providing an incomplete story. Here, we describe, in detail, an aggressive GBM that involved the subventricular zone in which normal stem cells reside in. The clinical characterization includes the patient’s clinical history, diagnosis, brain imaging studies, invasive surgery, and pathology. The molecular characterization of the resulting brain tumor stem cells includes in vitro, ex vivo and in vivo analyses. Taken together, our emphasis on research relevant to brain cancer patients covers an approach from clinical presentation to relevant laboratory research, which may narrow considerably a gap that exists between clinicians and basic research scientists. We have provided a comprehensive review of the cancer stem cell field, which may help design future therapies against brain tumors.

## Results

**Figure 1:**
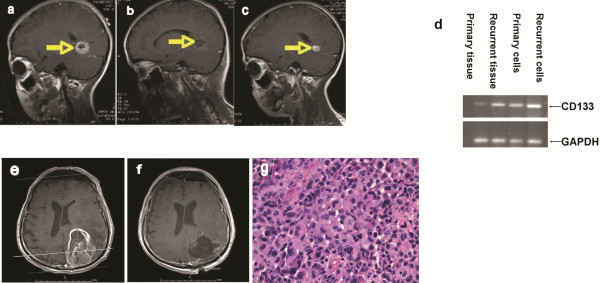
**Clinicopathological presentation of the brain tumor.** The child patient: MRI images show that primary tumor diagnostics, resection, recurrence of a child glioblastoma patient during treatment of surgery, radiation, and chemotherapy (Yellow arrow: Tumor mass): **a,** pre-operation (sagittal view), showing the characteristic appearance of the tumor; **b,** Immediate postsurgery (sagittal), showing the removal of the tumor; **c,** 3-month post-surgery (sagittal), showing recurrence of the tumor; **d,** agarose gel electrophoretic analysis of CD133 expression using RT-PCR. The adult patient: Pre- and post-operative magnetic resonance imaging (MRI) shows hemorrhage and involvement of the lateral ventricle (e, f). **e**: Pre-operative MRI (Gadolinum-enhanced) of previous intracerebral hemorrhage (before tumor operation). **f:** Post-operative MRI. **g:** Pathology photomicrographs showed typical glioblastoma multiforme with nuclear atypia, endothelial proliferation, and necrosis (hematoxylin and eosin stain). Necrosis was seen with the pseudopalisading pattern of malignant cells. This is a high-grade glia neoplasm, characterized by variably cellular patternless sheets of polygonal and fusiform cells with moderate to marked nuclear atypia, amphophilic cytoplasm, prominent nucleoli, and many mitotic figures. Irregular zones of geographic necrosis were surrounded by palisading neoplastic cells. The tumor was vascular with many blood vessels lined by plump endothelial cells interspersed within the glial component. The cellular areas of the neoplasm are merged gradually with adjacent cerebral cortex. Neuronal satellitosis was seen in the transitional zone. These are consistent with glioblastoma multiforme involving intraventricular zone.

As shown in Figure 1, the recurrent tumor showed higher CD133 expression than the primary tumor from the same young patient on both tumor tissue and cultured cell levels (Figure 1d). The result prompted us to hypothesize that the tumor residual CD133 positive cells may drive the tumor to recur. To address this hypothesis, we obtained a second tumor specimen from another patient to sort for CD133+ cells and followed up with comprehensive characterization, including imaging, surgical, pathological, molecular, cellular, and biological features.

### Imaging of the tumor before surgery

A **computed tomography** (**CT**) scan identified an area of heterogeneous soft tissue density in the left parietal lobe. There was a small ill-defined area of increased density in this region, which might represent hemorrhage. There was marked surrounding vasogenic edema and mass effect on the adjacent left lateral ventricle.

MRI of the brain, with contrast, showed a large heterogeneously ring-like enhancement within the left occipitoparietal lobe, measuring 6.0 x 4.5 cm and associated with marked edema (Figure 1e). There was a mild midline shift to the right by ~5.0 mm. There were also severe periventricular changes with increased signal. MRI images, obtained with gadolinium-enhancement, showed an early subacute stage of intracranial hemorrhage. There was left parietal hemorrhage measuring on the order of 3.7x3.3x2.1 cm, associated with vasogenic edema. These findings were consistent with those in the CT scan.

### Surgical therapy effectively debulked the tumor mass

A linear incision was made in the left parietooccipital region. Following craniotomy and dual incision, a plane was developed between the tumor and the cortical white matter, and circumferentially dissecting along the plane took place. Intraoperative specimens were sent for frozen section examination, confirming the diagnosis of malignant glioma (see below). Dissection was continued initially laterally and inferiorly, and fully developed a plane between the white matter and what appeared to be tumor. The medial dissection was carried to the falx, as directed by the MRI data. A deep plane and more superior plane in a circumferential manner following up the white matter and tumor plane were made. Bipolar electrocautery as well as suction were used following dissection. The occipital horn of the lateral ventricle on the left side was entered and an external ventricular drain was placed through the opening. Further inspection showed excellent hemostasis and gross total resection seemed to have been achieved.

Postoperative MRI (Figure 1f) showed surgical changes involving the left parieto-occipital lobe. There was a large cystic area identified at the operative site, as seen on the T1-weighted images. Surgical removal of the large, mixed, cystic (solid) mass in the left parieto-occipital lobe resulted in a fluid collection which measured 4.6 x4.9 cm at the operative site. There was a decrease in the amount of vasogenic edema and mass effect and a decrease in the shift of the midline toward the right as well as a decrease of the mass was seen on the left lateral ventricle.

### Pathological analysis determined high-grade glioma

Frozen section diagnosis of the left occipital brain tumor was consistent with malignant glioma. Microscopically, the occipital tumor showed a high-grade glial neoplasm (Figure 1g). It was characterized by variably cellular, patternless sheets of polygonal and fusiform cells with moderate to marked nuclear atypia, amphophilic cytoplasm, prominent nucleoli, and numerous mitotic figures (Figures 1g). Irregular zones of necrosis were surrounded by palisaded neoplastic cells. The tumor was vascular, with many blood vessels lined by plump endothelial cells interspersed within the glial component. The cellular areas of the neoplasm were merged gradually with nearby cerebral cortex; and neuronal satellitosis was noted within the transitional zone. A strong, positive, glial fibrillary acidic protein (GFAP) stain was noted.

### Tumor grew back after surgical and adjuvant therapies as monitored by CT and MRI

Two months after surgery, MRI of the brain, with & without contrast, showed that, within the region of the left posterior parietal lobe, there was a ring-enhancing cystic area measuring 4.5x3.05 cm. There was vasogenic edema associated with this ring-enhancing cystic area. There was extensive, abnormal, high signal intensity seen within the deep white matter and periventricular distributions bilaterally as well as within the right cerebral hemisphere. There was also increased signal seen within the thalamic region as well as within the internal capsule bilaterally.

Four months postsurgery, CT of the brain showed there was a prominent periventricular area of decreased attenuation. Postoperative changes were seen in the left posterior parietal area. There was a fluid collection noted. There were focal areas of encephalomalacia in the right and left cerebellum. There was ex vacuo dilatation of the posterior horn of the left lateral ventricle. The prominence of the ventricles and sulci was consistent with cortical atrophy. The patient passed away shortly thereafter (six months after the surgery).

### Cultured CD133-expressing cells behaved as cancer cells

**Figure 2:**
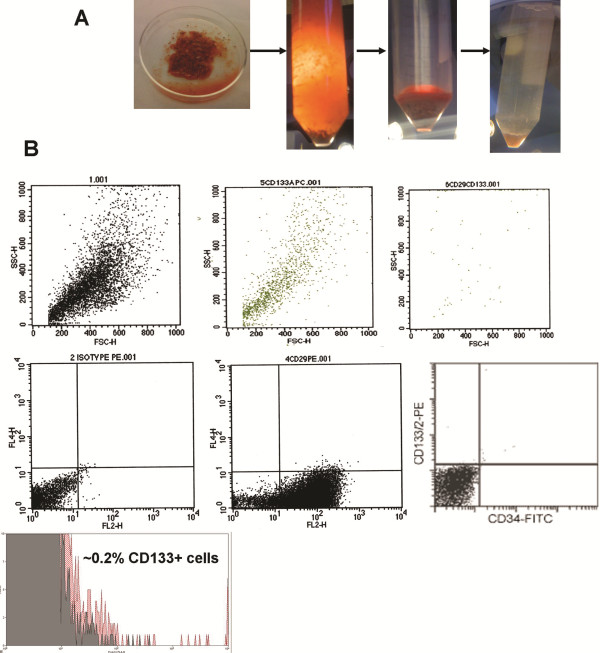
**Isolation and FACS of tumor cells. A.** Procedure for isolation and dissociation of tumor cells into single cells. The tumor specimens were minced by using crossed scalpels to cut them into small pieces over an ice-bath. The minced pieces were triturated with 50-mL and 25-mL pipette, consecutively. The sample was washed 6X with cold Hank’s buffer-saline solution (HBSS) without phenol red and allowed to settle by gravity (3–5 min). The supernatant was transferred to a fresh 50-mL conical polypropylene tube (Falcon, Becton Dickinson) and the precipitate (necrotic tissue [black] and vessel pieces) was discarded. The pieces were washed repeatedly until the supernatant became clear. Remaining red blood cells were removed by step-gradient centrifugation over Histopaque-1077. The pellet was red blood cells and the brain tissue was in the supernatant. The supernatant was washed with HBSS and centrifuged (183 g, 5 min, 3x) to remove the Histopaque-1077. The pellet was triturated sequentially with 10 mL, 5 mL, and 2 mL pipettes. The suspension was then digested with collagenases, papain, protease, DNase, and Dispase II. The loose cells were washed and the cell pellet was suspended in cell dissociation buffer. **B**. FACS analysis of tumor cells. The surface marker expression (CD133, CD29, CD34) were used. The antibodies were as for name/synonym/clone: CD29/integrin-β1/MAR4, CD34/Sialomucin-I/AC136, and CD133-1/Prominin-1/AC133.

**Figure 3:**
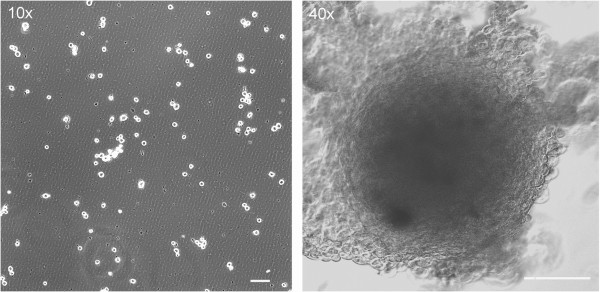
**Neurosphere formation was seen with phase-contrast microscopy of GBM CD133-positive tumor cell culture.** Single tumor cells were sorted for CD133 expression by magnetic bead cell sorting to collect CD133-positive cells. Single CD133-positive cells were then cultured. Neurospheres were seen when single CD133-positive tumor cells were cultured in EGF- and bFGF-containing, serum-free medium. The left panel is before culture of CD133+ cells (t: 0 day), and the right panel after six weeks (t: 42 days). (Left panel, scale bar = 50 μm; right panel, scale bar = 100 μm).

**Figure 4:**
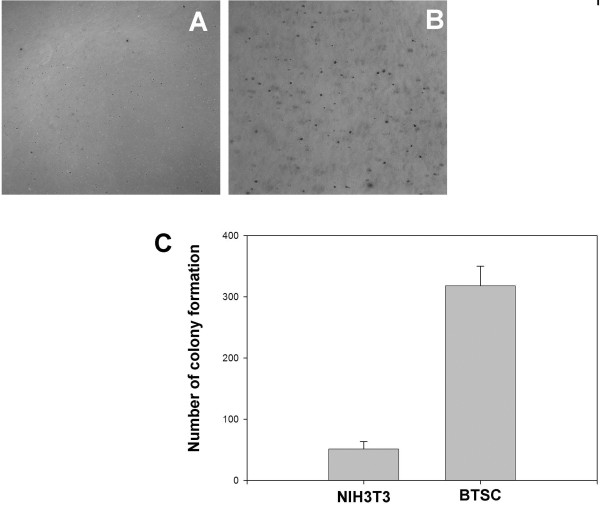
**Colonies were seen in clonogenic assay.** Growth of normal NIH 3 T3 cells (**A**) and CD133-positive tumor cells (**B**) in soft agar shown in micrographs by phase contrast microcopy (4x). **C**: The numbers of colonies were counted in a view field with the 4x objective at 14 days of culture and plotted in bar graphs from three independent repeats.

**Figure 5:**
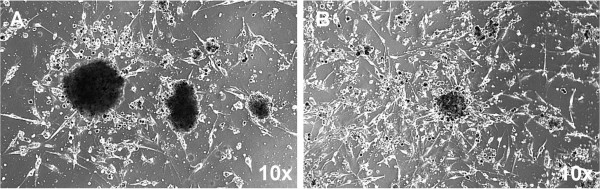
**Adherent culture on plastic dishes in serum-containing medium on fibronectin coated plates.** Neurospheres adhered to the plastic surface at day 1 (**A**) and spread out and grew at day 3 (**B**). A 10x objective was used for imaging.

**Figure 6:**
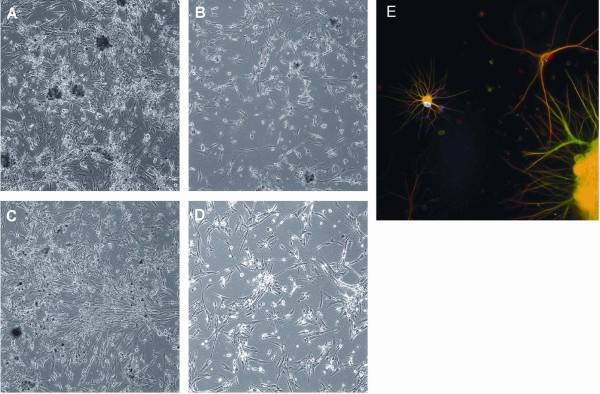
**Adherent culture in serum-containing medium on plastic dishes coated with extracellular matrices. A**: Coated with Matrigel™. **B**: Coated with collagen IV. **C**: Coated with laminin. **D**: Coated with fibronectin. **E**: Strong, positive, GFAP stain was noted (in red). Immature neurons positive for β-Tubulin III (green) were around the neurosphere (lower right) when grown on Matrigel™ in serum-containing medium. Nuclei were stained blue by Hoechst. (**A**, **B**, **C**, **D**: 10x objective; **E**, 20x objectives). The conclusion for panels **A**, **B**, **C**, **D** is that the cells behave differently in different adhesion molecules coated dishes. They grew in different speeds: from fast to slow – Matrigel™ (A) → Laminin (C) → Collagen IV (B) → Fibronectin (D).

A relatively morphologically-homogeneous tissue was obtained after the differential purification procedure (Figure 2A), from which single cells were obtained containing ~0.2% CD133-positive cells (Figure 2B). The recurrent tumor showed higher CD133 expression than the primary tumor from the same patient (Figure 1d). Single cells were grown into neurospheres under stem cell culture technique (Figure 3). The control was normal NIH3T3 mouse fibroblasts, grown in parallel, which ceased dividing whereas CD133-positive cells continued to proliferate under the otherwise restrictive conditions of soft agar (Figure 4). Although the CD133-positive cells formed colonies in soft agar with similar efficiencies (80–100%), the sizes of the colonies varied widely, suggesting they were heterogeneous (Figure 4). There was little colony formation with NIH3T3 cells. The CD133-positive neurospheres adhered to fibronectin in serum-containing medium and spread out (Figure 5) and extended neurite-like processes (Figure 6). These cells expressed certain differentiation markers, such as GFAP and β-Tubulin-III (Figure 6E). The cells preferred certain adhesion molecules (Figure 6). They grew from fast to slow – Matrigel™ (A) → Laminin (C) → Collagen IV (B) → Fibronectin (D). Cells grew faster with Matrigel™ (a gelatinous protein mixture secreted by Engelbreth-Holm-Swarm mouse sarcoma cells) than with any other single adhesion molecule presumably because Matrigel ™ resembles the complex extracellular environment found in many tissues that contains multiple species of adhesion molecules (Laminin, Entactin, and Collagen) and growth factors as well as other components. Matrigel™ has been used to maintain the pluripotent, undifferentiated state (self-renewal) and promote stem cell growth and differentiation upon dilution [27].

**Figure 7:**
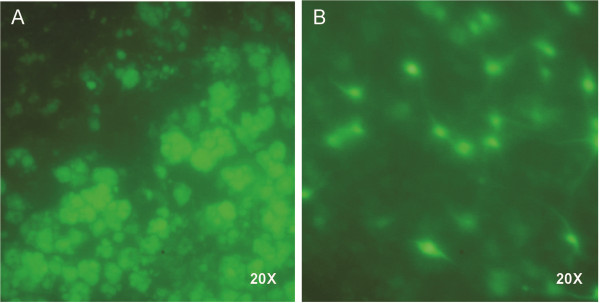
**Normal neural stem cells and brain tumor stem cells behaved differently on the organotypic brain slice.** All cells were labeled with Lentivirus-GFP and underwent live cell imaging. **A**: CD133-positive GBM stem cells clumped together. **B**: Normal neural stem cells spread out and extended processes. (All 20x objective).

It has been shown that tissue elasticity regulates stem cell morphology and their lineage specification [28]. On plastic Petri dishes, the CD133+ cells spread out in culture (Figures 5 and 6); however, these dishes provide only an artificial environment. To address this issue, we used an ex vivo organotypic brain slice culture system that allows the CD133-positive cells to grow in cell clumps in the brain-mimicking environment (Figure 7A) while normal neural stem cells spread out to be single cells and underwent extended processes (Figure 7B). The CD133-positive cells, therefore, behaved as they did in soft agar (clonogenic formation) as described above and as they did after in vivo transplantation as described below.

### Diverse marker expression

**Figure 8:**
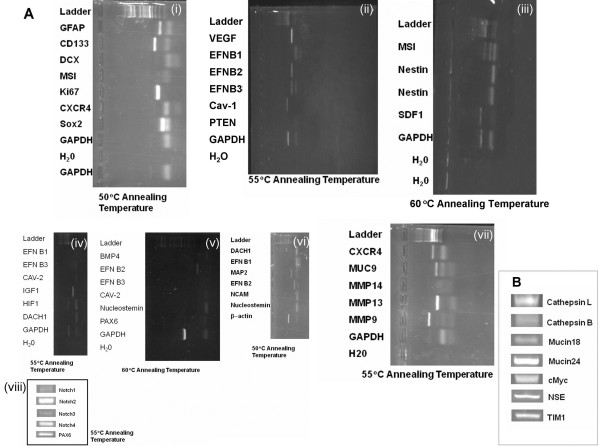
**Agarose gel electrophoretic analysis of biomarker expression using RT-PCR.** Panel **A** (sub-panels i – viii): Biomarker expressions of GBM tumor cells derived from CD133-positive cells grown in EGF- and bFGF-containing, serum-free medium (Note IGF1 should be IGF1R in Figure 8-iv. MAP2 should be MMP2 in Figure 8-vi). Panel **B**: Biomarker expressions of tumor cells differentiated from GBM with serum-containing medium.

The CD133+ cells were assayed for expression of well-established genetic biomarkers for neural stem cells and differentiated neural cells using RT-PCR under different annealing temperatures (Figure 8; Table 1). Medium-level expression of stem cell markers included Nestin, Notch 4, Cav-1, Nucleostemin, EFNB2, EFNB3, and HIFα1 (Figure 8A). Low-level expression of Musashi, DACH1, Notch 1, Notch 3, Cav-2, EFNB1, and EFNB3 was also seen (Figure 8A). The high-level expression genes consisted of CD133 (i), Ki67 (i), MMP13 (vii), Sox2 (i) and Notch2 (viii). We observed that proteoglycans were expressed in the cells cultured in serum-containing medium. Low-level expression biomarkers from the cells in serum-containing medium consisted of Mucin 18 and Cathepsin B (Figure 8B). Medium to high-level expression genes included c-Myc, neural specific endolase (NSE), Mucin 24, TIMP1, and Cathepsin L (Figure 8B). Tumor suppressors and oncogenes (p53, PTEN, c-Myc) were also found to be present in these tumor cells. Some of these biomarkers in the tumor stem cells were found in the side-by-side control normal neural stem cells, including those genes described previously from our group [29].

**Table 1:** Summary of marker expression determined by RT-PCR

**Stem cell markers**		**Proliferation, angliogenic, migration markers, growth factors, adhesion**	
SOX2	++++++	Ki67	+++++
DACH1	+	VEGF	+++
CD133	++++	HIF1	+
Nestin	+++	CXCR4	++
Nucleostemin	++++	SDF1	+
		IGF1R	++
		NCAM	+++
**Glial Cells**		PAX6	++++
GFAP	++		
		**Ephrins receptors and ligands (cell repulsion, adhesion, migration)**	
**Neuronal**		EphB1 Receptor	n/a
DCX	+	EphB2 Receptors	++++++
MSI	+	EphB3 Receptors	+++++
MAP2	+	EFNB1\	+
Neural Specific Endolase	++++	EFNB2	++
Beta-3Tubulin III	+	EFNB3	+
		**MMPs, Peptidases**	++
		MMP2	+++
**Tumor Suppressors/Oncogenes**		TIMP1	
p53	+	MMP9	+++
PTEN	+	MMP13	++++
Bmi-1	++	MMP14	+
CAV-1	++	Cathepsin B	+
CAV-2	+	Cathepsin L	+++
c-Myc	+++		
		**Controls**	
**Proteoglycans**		GAPDH	+++
Mucin9	++	Beta-Actin	++
Mucin18	+	H_2_O	
Mucin24	++++		
**Notch-Family**			
Notch1	+		
Notch2	+++		
Notch3	+		
Notch4	++		

Low-level expression is designated as “+” while medium level expression is designated as “++” to “+++++” and the highest level is designated as “++++++”.

### Caveolin-1 is expressed in the CD133-positive cells

**Figure 9:**
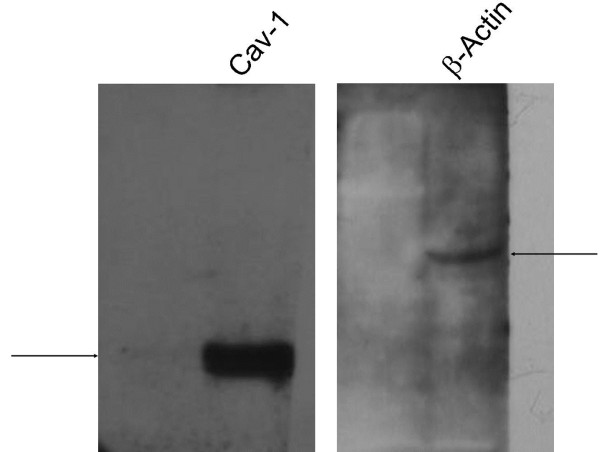
**Western blot analyses of GBM cells cultured with serum-free medium.** Cells were grown as described in Methods section and the lysate proteins were subjected to SDS-PAGE. The sample was subjected to immunoblot analysis with caveolin-1 mouse monoclonal antibody (4 H312, sc-70516; Santa Cruz Biotech) probe. Note that anti-Caveolin-1 mAb specifically binds to Caveolin-1 isoforms (21–24 kDa) while anti-β-Actin antibody specifically recognizes β-Actin (42 kDa). These results further demonstrate that Caveolin-1 is present at both the protein level and mRNA level (Figure 8).

We have observed, for the first time, that Caveolin-1 mRNA is expressed in CD133-positive cells (Figure 8A). Caveolin-1 is a well-established cancer marker for breast cancer prognostics. We confirmed that consistent with mRNA, Cav-1 protein was expressed in the CD133+ tumor cells by Western blot analysis (Figure 9). Both Cav-1α and Cav-1β isoforms were expressed in these cells (data not shown), as doublets which previously described in other types of normal cells [30].

### CD133-positive cells formed brain tumors in vivo

**Figure 10:**
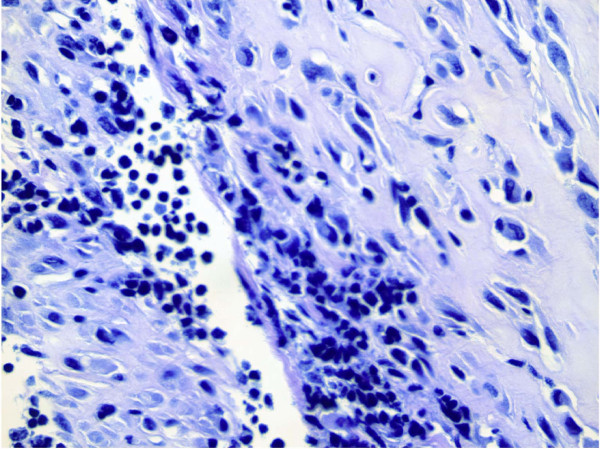
**Photomicrograph of the GBM-derived tumor that arose following stereotactic intracranial transplantation of CD133-positive tumor cells.** High power view of transplanted human CD133-positive cells grown in the brain of an immune-deficient NOD/SCID mouse is shown (40x objective). The tumor has a solid growth pattern. The tumor cells have darkly stained nuclei and scant cytoplasm. Nucleoli are not easily identified. Tumor necrosis and vascular proliferation are evident similar to the patient tumor shown in Figure 1B.

To prove the patient’s tumor-derived CD133-positive lineage was capable of forming a tumor, we performed stereotactic transplantation of CD-133-positive cells into the brains of immune-deficient NOD/SCID mice. The resulting tumor histology showed nuclear pleomorphism and high mitotic activity (Figure 10), which strongly resembled the histological features of the patient’s original glioblastoma (Figure 1g). All these data combined, therefore, strongly suggested that CD133-positive cells isolated from the GBM tissue mass were cancer stem cells (CSCs).

## Discussion

In this report, we have included: 1) a detailed clinical course, 2) radiological findings, 3) the surgical approach and its results, 4) pathological details, 5) marker expression analysis of tumor cells derived from the CD133-positive cells, and 6) evidence for ex vivo and in vivo behavior including tumor-initiating capacity. Clinically, it is of great interest to have a successful isolation of glioblastoma stem cells from a rare GBM that involves the neurogenic ventricular wall. We have found in this rare case that a tumorigenic CD133-positive progenitor cell phenotype is part of the tumor. The mRNA expression of an array of heterotypic biomarkers may explain the course of this patient's clinical outcome as gene expression indicates the participation of unique cancer-related transcripts specifically related to GBM stem cells, such as caveolin-1 and −2. Their expression in GBM CSC has not been previously reported in the literature.

GBMs usually form in the cerebral white matter, grow quickly, and can become large before producing symptoms. Malignant tumor cells infiltrate from primary tumor sites to nearby tissues, representing the major cause of death in patients. In the clinic, the intrinsic infiltration of single glioma cells into brain parenchyma renders these cancers resistant to the current treatment of surgical removal in combination with radiation-, chemo- and immuno-therapies [31]. Invariable infiltration into adjacent brain parenchyma, crossing commissures to expand to the opposite cerebral hemisphere, is a hallmark of the malignancy of GBM. Thus, despite recent advances in surgical and medical therapy, the prognosis for patients diagnosed with high-grade GBM remains poor. The realization that a self-replication mechanism may be shared by both normal stem cells and cancer cells has led to the new concept of the cancer stem cell (CSC) [6,32]. Similar mechanisms may control normal and cancer stem cell properties. This concept as has been supported by reports that showed the existence of a cancer stem cell population in human brain tumors of both children and adults with different phenotypes [33-35]. Both normal and tumor stem cell populations are heterogeneous with respect to proliferation and differentiation. The difference between normal neural stem cells and tumor stem cells has not been fully defined [7,36], but it has been speculated that brain tumor stem cells may be a cause of the resistance of tumors to conventional treatments, and high recurrence rate [37-40]. However, targeted elimination of tumor stem cells may be detrimental if it also eliminates normal neural stem cells. In our study, glioblastoma stem cells from a rare GBM that involves the neurogenic ventricular wall may tackle and hijack the source of the normal neural stem cells that reside in neurogenic ventricles.

The hallmark of the malignant glioblastoma is its diverse marker expression. Marker expression in the prognosis of malignant brain tumors has been explored, the main issue being the heterogeneous expression of most of the genes examined [41-50]. We have presented evidence of the successful isolation and characterization of a small subpopulation of cancer stem cells. The molecular features of these tumor cells may provide potential new therapeutic targets, and therefore strategies that may control them. Certain molecular markers are consistent with those previously reported [51]. For example, Murat and colleagues (2008) provided the first clinical evidence for the implication of high epidermal growth factor receptor (EGFR) expression associated with resistance to concomitant chemoradiotherapy in a “glioblastoma stem cell" or "self-renewal" phenotype [40].

The clongeneity of these single CD133 positive cells showed biological differences in the growth capacity as shown in Figure 4 and Figure 7. In fact, Dr. Cavenee and Dr. Furnari and colleagues showed that CSCs undergo clonal evolution from a single GBM cancer stem cell to extensive heterogeneity at the cellular and molecular levels [52]. The single-cell generated heterogeneity confers a biological advantage to the tumor by creating an intratumoral and tumor-microenvironment community that serves to maintain the heterogeneous tumor composition and to promote tumor growth. This tumor community allows interactions between CSCs and/or tumor cells and their environment and between different CSCs and /or tumor cell subclones. Those interactions need to balance out. An inbalance may drive tumor growth, drug resistance, immune suppression, angiogenesis, invasion, migration, or more CSC renewal. We suggested that a delicate balance may be modulated by innovative therapeutics to keep the tumor in surveillance check [26]. We thought that in the context of stem cell development, there is a parallel with the concept of quiescent or dormant cancer stem cells (CSCs) and their progeny, the differentiated cancer cells; these two populations communicate and co-exist. The mechanism with which determines to extend self-renewal and expansion of CSCs is needed to elucidate.

CD133 (prominin-1), a neural stem cell (NSC) marker implicated in brain tumors, notably glioblastoma, was highly expressed in our material. Interestingly, CD133 is also expressed in the glioma cell lines U251 and U87MG [53]. Remarkably, a recent study showed that the level of membrane particle-associated CD133 is elevated in early stage glioblastoma patients and decreases dramatically in the final stage of the disease [54]. This change may be used for diagnosing and surveying glioblastoma initiation and progression [55,56]. More clinically relevant, CD133 is associated with specific extracellular membrane particles in cerebrospinal fluid, which can be routinely used for diagnosis and prognosis in neurological diseases. Malignant brain tumors have a higher CD133 index than low-grade tumors [57]. Purified populations of CD133-positive tumor cells injected into the brains of NOD/SCID mice induced tumors that were heterogeneous and had the characteristic of infiltration [58,59]. It has also been shown that transplantation of neurospheres derived from glioblastoma tumor cells cultured in EGF and bFGF-containing media drove tumor formation in immune-deficient mouse models [60,61]. These CD133-positive tumor cells may be a leading force for reinitiating tumor genesis and progression [62]. However, there is debate about the lineage relationship between normal NSCs and brain cancer stem cells. It is not yet fully understood if CD133-positive brain CSCs are derived from CD133-positive normal NSCs. Thus, it is still questionable if tumor therapies can be developed for targeted destruction of CSCs without damaging normal NSCs. Dr. Bota and colleagues have recently found that both the proteasome inhibitor bortezomib (BTZ) and the epidermal growth factor receptor tyrosine kinase inhibitor erlotinib (ERL) decreased glioma stem-like cells (GSCs) proliferation but not NSC viability [63]. Surprisingly, commonly used temozolomide (TMZ) and cisplatin (CIS) were more toxic for NSCs than for GSCs. This in vitro observation may inspire a new journey to search for GSC-specific destruction agents, which are not detrimental to NSCs.

Angiogenesis is a critical component of brain tumor growth. Consistent with our pathological findings, VEGF is highly expressed, confirming that neovasculization is driven by the up-regulation of VEGF around tumors. Recent clinical trials of antivascular endothelial growth factor agents for glioblastoma show promising progression-free and better overall survival rates, even without inhibiting tumor growth [64].

The intermediate filament protein, Nestin, and the RNA-binding protein, Musashi, are expressed by NSCs during CNS development. Their expression in glial tumors correlated with the levels of Cysteine Cathepsins [65] that are known as prognostic markers of several tumors [41]. Nestin is a strong prognostic marker of glioma malignancy; the invasive cells may well be closely related to glioma stem cells [41], which our data confirms. Nestin functions in the organization of the cytoskeleton, cell signaling, organogenesis, and cell metabolism. It is down-regulated in mature cells, whereas GFAP, neurofilaments, and PDGFR are expressed in differentiated astrocytes, neurons, and oligodendrocytes, respectively [66]. Neoplastic transformation up-regulates Nestin expression in astrocytes of the adult CNS, suggesting that its reactivation may relate to tumor genesis [67]. Nestin has been shown to be a strong prognostic marker for glioma malignancy and its expression correlates with patient survival [68]. We have found Nestin expressed in both CD133-positive tumor cells and differentiated tumor cells, although the latter with down-regulation, which suggests the existence of residual neural stem cells after induced differentiation.

Peptidases hydrolyze macromolecular components of the extracellular matrix, support the malignant invasive behavior of brain tumor cells, and promote brain tumor progression by advancing tumor angiogenesis [69-71]. Peptidases consist of matrix metalloproteinases (MMPs), Cathepsins, and Plasminogen activators. Among MMPs, MMP2 and MMP9 strongly correlate with glioma progression [72-74]. Most importantly, Wong and colleagues found that increased cerebrospinal fluid (CSF) MMP-9 activity could be a biomarker of disease activity in patients with malignant gliomas, before any changes are detectable on MRI [75]. Lysosomal Cathepsin B is highly expressed in malignant glial cells and endothelial cells of vascularized glioblastoma, an indication of a shorter survival time. Besides invasion, Cathepsin L may play a role in decreased susceptibility of anaplastic glioma cells to apoptosis [76,77]. Cathepsin B has been considered a marker for malignancy in the more aggressive type of meningiomas [78]; developing inhibitors of these peptidases might help control local spread [70,77].

Originally identified as an oncogenic partner of c-Myc in murine lymphoma genesis, Bmi-1 is a member of the polycomb group transcriptional repressors [79,80]. Bmi-1, a proto-oncogene for inhibition of p53 involved in cell cycle and self-renewal, is required for the postnatal maintenance of stem cells in multiple tissues, including the central nervous system (CNS) and peripheral nervous system (PNS). Bmi-1 was highly expressed in the GBM tumor cells we cultured from our case, consistent with a previous report [34]. Targeting of the Bmi-1 in stem cells by microRNA-128 inhibits glioma proliferation and self-renewal, implying that miRNA-128 may be a therapeutic target agent for the "stem cell-like" characteristics of glioma [81].

Finally, we have found that Caveolin-1 and Caveolin-2 are expressed in our CD133-positive lineage (Figure 8, Figure 9). Interestingly, their expression in GBM CSCs has not been previously reported in the literature. Rather, this has been reported in commercialized glioma non-stem cell lines, such as glioblastoma cell line U87MG [82]. However, their clinical significance in brain tumor diagnosis and prognosis remains to be determined. Caveolin-1 has been found in detergent-resistant plasma membrane microdomains involved in signaling transduction in many cell types, including neurons and astrocytes [83-85]. It is a secreted biomarker in some pathological conditions [86]. In prostate cancer, high preoperative serum Caveolin-1 levels have been established as a biochemical predictor of cancer progression and recurrence [87], suggesting a poor prognosis (shorter time to cancer recurrence). Lisanti’s group analyzed breast tissue samples from 154 women diagnosed with breast cancer using immunohistochemical staining of stromal Caveolin-1 [88]. Among each subgroup of patients, as grouped by prognostic factors such as hormone status, disease stage or lymph node status, a loss of stromal Caveolin-1 remained the strongest single predictor of breast cancer patient outcome. Progression-free survival (PFS) was also affected by the loss of stromal caveolin-1. The approximate 5-year survival rate for patients positive for stromal Caveolin-1 was 80% vs. 7% for patients negative for stromal caveolin-1, i.e. a ~11.5-fold reduction in 5-year PFS. Caveolin-1 serves not only as a prognostic marker, but also as a means of therapeutic stratification. Caveolin-1 can be detected at breast cancer diagnosis, which is important because high-risk patients would benefit from more aggressive antiangiogenic therapy. A prognostic biomarker present in the stroma rather than the epithelial cancer cell is a paradigm-shift, since a diagnostic test may not require DNA-based technologies for cost-effective identification for high-risk breast cancer patients at diagnosis.

Despite their clinical importance, little is known about the underlying composition and cellular interactions of tumors that govern their degree of malignancy, and consequently, provide targets to control their growth. The diverse biomarker expression reflects the nature of heterogeneity in the tumor, a mixture of cells at different stages of their development. Indeed, Vescovi’s group discovered that at least two types of CSCs bear quite diverse tumorigenic potential and distinct genetic anomalies, yet derive from common ancestor cells within different regions of the same human GBM [89]. Thus, therapeutic success relies on an effective strategy to select for a therapy to target some particular stage of tumor cell development at which tumor cells are most susceptible to treatment.

The transition from neural stem cells to cancer cells [25] may be activated by expression of some cancer driver, characteristic of dominant clones (single cells), but not in every cell [26]. Cancer cell phenotypes may be derived from such a few dominant single cells with a continuum from single driver stem cells to cancer cells. We may need to define at what point we call it a cancer cell, for which a treatment is needed. Such a point of time in cancer development, namely the therapeutic window [90], may be defined by an integrated genomic [91] and epigenomic [92,93] analyses through applying next-generation sequencing technology. However, the current whole-genome sequencing mainly on the bulk tumor that also includes stromal and immune cells, does not specifically address the tumor-initiating cells (or CSCs). Developing therapeutic window-specific drugs may be realized by using patient-specific cancer stem cell lines for chemical and genetic screens as described previously [94]. We need to focus on these tumor-initiating cells at a single-cell level. Glioma stem cell lines derived from patients like the one described in our study may be used for single cell analyses.

## Conclusions

The tumor-forming, CD133-positive cancer stem cells (CSCs) identified from a brain tumor involving the neurogenic lateral ventricular wall may drive the rapid recurrence of the tumor. Determination of mechanisms which enhance self-renewal and expansion of the CSCs may help elucidate novel therapeutic strategies specific control of tumors.

## Methods

### Patient’s background

The enrolled patient gave written informed consent to the surgical and experimental procedures as well as to publications of this case report and any accompanying images. The protocol and consent were approved by our Institutional Review Board.

History of present illness: An adult, left-handed, white male had complained of progressive right-sided weakness as well as a decrease in mentation. Serial computed tomographic (CT) imaging showed persistent edema in the left parietofrontal region, with a left parietal intracerebral hemorrhage. Over four weeks, he had decreased mentation and speech. His right side also became much weaker. The neurological examination showed facial weakness, right worse than the left. Motor examination showed right-side poor coordination with pronator drift and about 2/5 motor strength (left side was 4 to 4+/5). Sensory systems appeared to be intact, but he was hyporeflexic throughout.

CT scan of the brain without contrast, two weeks after presentation, showed extensive edema that appeared as a hypodense area. The hypodensity had increased in size in the left region as confirmed with magnetic resonance imaging (MRI).

### Surgery

Stereotactic craniotomy was performed and the left-side ventricle occipital horn tumor was debulked. There were no complications with the procedure.

### Tumor histology

Tumor samples were obtained during surgery. Formalin-fixed, paraffin-embedded tissue blocks were prepared from the tumor specimen and hematoxylin and eosin–stained sections were reviewed by certified pathologists.

### Tumor cell culture

Some of the tumor was used for live cell isolation. The procedure for isolation of neural progenitor cells was followed as described previously by us [29,95,96] and others [34,57,97], with an added step for clearing red blood cells and necrotic cells [98]. Briefly, tumor specimens were minced by using crossed scalpels to cut them into small pieces over an ice-bath. The minced pieces were triturated with 50-mL and 25-mL pipette, consecutively. The sample was washed 6X with cold Hank’s buffer-saline solution (HBSS) (Invitrogen, Carlsbad, CA) without phenol red and allowed to settle by gravity (3–5 min). The supernatant was transferred to a fresh 50-mL conical polypropylene tube (Falcon, Becton Dickinson) and the precipitate (necrotic tissue [black] and vessel pieces) was discarded. The pieces were washed repeatedly until the supernatant became clear. Remaining red blood cells were removed by step-gradient centrifugation (183 g, 5 min) over Histopaque 1077 (Sigma, St. Louis, MO) [98]. The pellet was red blood cells and the brain tissue was in the supernatant. The supernatant was washed with HBSS and centrifuged (183 g, 5 min, 3x) to remove the Histopaque-1077. The pellet was triturated sequentially with 10 mL, 5 mL, and 2 mL pipettes. The suspension was then digested with collagenases, papain, protease, DNase, and Dispase II. The sample was washed and the cells were triturated with 1-mL pipette. The loose cells were suspended in cell dissociation buffer (Invitrogen, Carlsbad, CA).

Part of the above cells were analyzed by flow cytometry using a Becton Dickinson FACS Calibur (Franklin Lakes, New Jersey, USA) for surface marker expression (CD133, CD29, CD34). All the antibodies used in this study were obtained from BD Pharmingen (name/synonym/clone: CD29/integrin-β1/MAR4, CD34/Sialomucin-I/AC136, and CD133-1/Prominin-1/AC133). The rest of the cells were sorted by magnetic-activated cell sorting (MACS) with the Indirect CD133 MicroBead Kit (Miltenyi Biotec GmbH, Germany).

Viability of single cells was determined using the fluorescein diacetate (FDA)/propidium iodide (PI) assay [99-101]. For serum-free cell culture, 4x10^4^ CD133-positive cells were resuspended in 5 ml of DME/F12 containing 10% BIT 9500 supplement (Stem Cell Technology), 1x N2 supplement, 20 ng/mL EGF, 20 ng/mL bFGF, 2 μg/mL heparin plus an antibiotic cocktail and plated into an uncoated 60-mm dish where they formed neurospheres. The antibiotic cocktail contained 10,000 U/mL penicillin G, 10,000 μg/mL streptomycin sulfate, 2.5 μg/mL amphotericin B, 10 μg/mL gentamicin sulfate, and 10 μg/mL ciprofloxacin (antimycoplasma). Part of the cells were grown in extracellular matrix-coated plates with serum-containing culture medium (Advanced-DME [Adv-DME]; Invitrogen, Carlsbad, CA) containing 5% FBS plus the antibiotic cocktail to induce differentiation. The extracellular matrices used for coating plates included collagen IV, fibronectin, laminin, and Matrigel. Part of CD133+ cells was cultured in 96-well plate for single-cell culture to form single cell-derived neurospheres.

### Clonogenic assay

The clongenic assay used was described previously [102]. Briefly, for testing cell growth in soft agar, 10^3^ cells dissociated from neurospheres were suspended in 3 ml Adv-DME containing 5% FBS and 0.33% Sea Plaque low-melting-temperature agarose (American Bioanalytical, Natick, MA, USA). The cells were then plated onto 60-mm plates over a 2-ml layer of solidified Adv-DME containing 5% FBS and 0.5% agarose, and allowed to settle to the interface between these layers at 37°C. After 20 min, plates were allowed to harden at room temperature for 30 min before being returned to 37°C. The plates were fed every 3–4 days by overlaying with 2 ml of medium containing 0.33% agarose. After 2 weeks, the plates were stained with 0.1% crystal violet in 50 (vol/vol) Methanol. Plates were destained with cold water. Colonies were photographed under 4x magnification and counted. Multiple plates were used for statistical analyses. NIH 3 T3 cells were used as a control.

### Preparation of organotypic slices from murine brain tissue

Animal protocols were approved by the IACUC. Organotypic brain slices (OTS) were prepared from 8-17-day-old neonatal mice (CD-1, Charles River, Wilmington, MA) by modifying our previously published procedure [103]. Briefly, mice were euthanized in a CO_2_ chamber and then sterilized with a 70 alcohol solution. After cardiac perfusion with saline solution, the mouse was decapitated with surgical scissors and brains were removed with surgical knives and tweezers and placed in Adv-DME on ice. Each brain was then embedded in 4 LMT agarose (Invitrogen), and glued to the cutting stage of the vibratome (VT100S, Leica, Wetzlar, Germany). Slices ranging between 200–300 μm in thickness were generated with the vibratome and washed 3 times in HBSS to remove any tissue debris and any potentially toxic substances (e.g. excitatory amino acids). The slices were then placed on culture plate inserts (0.4 μm Millicell-CM, Millipore) in sterile-filtered slice culture medium (SCM). SCM was prepared by mixing 50 Minimal Essential Medium (Invitrogen), 25 heat-inactivated horse serum (Invitrogen, Carlsbad, CA), 25 mM HEPES, 25 HBSS, 6.4 mg/ml glucose, 0.5 mM glutamine, 10 ng/mL of insulin-like growth factor (IGF), and 1 penicillin-streptomycin-glutamine (Invitrogen). One mL of SCM was added to each OTS culture and the OTS was incubated at 37°C and 5 CO_2_.

#### Transplantation of cells onto organotypic brain slices

After 2 days in culture, the OTS was gently washed three times with SCM. CD133-positive cells or neural stem cells (SC27, Refer to [29]) were labeled with a lentivirus construct carrying the GFP gene (Gift from Dr. Wange Lu, University of Southern California). The GFP-labeled cells (200 ~ 10^3^ cells in 0.2 μL) were deposited onto the surface of the OTS. After 6 hours, the slices were washed with SCM to remove unattached cells. Cells engrafted in a week and differentiated in 4 to 7 weeks on OTS.

### Semi-quantitative RT-PCR

The method and primers used specifically for stem cells were previously described by us [104]. Briefly, 1 μg of total RNA was subjected to RT-PCR. Twenty-five rounds of an amplification cycle of 94°C for 30 s, 57°C for 30 s, and 70°C for 30 s were used in PCR reactions in a 2720 Thermal Cycler from Applied Biosystems (Foster City, California, USA). All the primers used are shown in Table 2 and are as described previously [104].

**Table 2:** Primer design: oligo nucleotide sequences

**Gene**	**Sequences (5' to 3')**
*Beta-Actin F*	GCACCACACCTTCTACAATGAGC
*Beta-Actin R*	TTGAAGGTAGTTTCGTGGATGCC
*Beta-tubulin III F*	AACGAGGCGCTCTACGACATC
*Beta-tubulin III R*	CTCCTCCTCGTCGTCTTCGTA
*Bmi-1AS*	CATTGCTGCTGGGCATCGTAAG
*Bmi-1 S*	GGAGACCAGCAAGTATTGTCCTTTTG
*BMP4 F*	GTGAGGAGCTTCCACCACGA
*BMP4 R*	ACTGGTCCCTGGGATGTTCTC
*Cathepsin B F*	GCAGCCTCAGCCACCCAGAT
*Cathepsin B R*	CCACCATTACAGCCGTCCCCACAC
*Cathepsin L F*	CCGGGGAGGGCAGTTGAG
*Cathepsin L R*	CCTTGAGGCCCAGAGCAGTC
*Cav-1 F*	GGACATCTCTACACCGTTCCC
*Cav-1 R*	TTATATTTCTTTCTGCAAGTT
*Cav-2 F*	ATGGGGGCTGGAGACGGAGAAG
*Cav-2 R*	TCAATCCTGGCTCAGTTGCAG
*CD133 AS*	ACGCCTTGTCCTTGGTAGTGTTG
*CD133 S*	CTGGGGCTGCTGTTTATTATTCTG
*c-Myc F*	AAGGTCAGAGTCTGGATCAC
*c-Myc R*	TAACTACCTTGGGGGCCTTT
*CXCR4 F*	CACCGCATCTGGAGAACCA
*CXCR4 R*	GCCCATTTCCTCGGTGTAGTT
*Dach1 S*	AGGCTTTCGACCTGTTCCTGAA
*Dach1 AS*	GCTGTCAGACCTGTTGGTGGAA
*DCX AS*	GTTTCCCTTCATGACTCGGCA
*DCX S*	AATCCCAACTGGTCTGTCAAC
*EPHB1 S*	GAGATGGACAGCTCCAGAGG
*EPHB1 AS*	CCAGCATGAGCTGGTGTAGA
*EPHB2 S*	AAAATTGAGCAGGTGATCGG
*EPHB2 AS*	TCACAGGTGTGCTCTTGGTC
*EPHB3 S*	AGCAACCTGGTCTGCAAAGT
*EPHB3 AS*	TCCATAGCTCATGACCTCCC
*EFNB1 S*	GGAGGCAGACAACACTGTCA
*EFNB1 AS*	GAACAATGCCACCTTGGAGT
*EFNB2 S*	GCAAGTT-CTGCTGGATCAAC
*EFNB2 AS*	AGGATG-TTGTTCCCCGAATG
*EFNB3, S*	CTGAAATGCCCATGGAAAGA
*EFNB3, AS*	ACGCCCAGCAAGAGCAGCGC
*GAPDH S*	ACCACAGTCCATGCCATCAC
*GAPDH AS*	TCCA CCACCCTGTTGCTGTA
*GFAP S*	ACATCGAGATCGCCACCTAC
*GFAP AS*	ACATCACATCCTTGTGCTCC
*HIF1 S*	GTCGGACAGCCTCACCAAACAGAGC
*HIF1 AS*	GTTAACTTGATCCAAAGCTCTGAG
*IGF1R S*	ACGCCAATAAGTTCGTCCAC
*IGF1R AS*	TCCATCCTTGAGGGACTCAG
*Ki67 F*	GGAGGCAATATTACATAATTTCA
*Ki67 R*	CAGGGTCAGAAGAGAAGCTA
*MMP2 F*	CCA CGT GAC AAG CCC ATG GGG CCC C
*MMP2 R*	GCA GCC TAG CCA GTC GGA TTT GAT G
*MMP9 F*	CAACATCACCTATTGGATCC
*MMP9 R*	CGGGTGTAGAGTCTCTCGCT
*MMP13 F*	TGCTCGCATTCTCCTTCAGGA
*MMP13 R*	ATGCATCCAGGGGTCCTGGC
*MMP14 F*	CGCTACGCCATCCAGGGTCTCAAA
*MMP14 R*	CGGTCATCATCGGGCAGCACAAAA
*msi1 S*	GAGACTGACGCGCCCCAGCC
*msi1 AS*	CGCCTGGTCCATGAAAGTGACG
*Mucin9 F*	CATGACCGGTGGACTTTTCT
*Mucin9 R*	TCCTGTGAACCTTTCCCAAC
*Mucin18 F*	GTGTTGAATCTGTCTTGTGAA
*Mucin18 R*	ATGCCTCAGATCGATG
*Mucin24 F2*	GTTAATACTACCTGCTTTTGGATAGAATGT
*Mucin24 R2*	CCACTTGACAATCACTAACTGTTGAG
*NCAM1 AS*	GGTGTTGGAAATGCTCTGGT
*NCAM1 S*	AGGAGACAGAAACGAAGCCA
*Nestin*	GGCAGCGTTGGAACAGAGGTTGGA
*Nestin R*	CTCTAAACTGGAGTGGTCAGGGCT
*Notch1 F*	TGTTAATGAGTGCATCTCCAA
*Notch1 R*	CATTCGTAGCCATCAATCTTGTCC
*NSE AS*	GACAGTTG CAGGCCTTTTCTTC
*NSE S*	CATCGA CAAGGCTGGCTACACG
*Nucleostamin F*	CATGACCTGCCATAAGCGGT
*Nucleostamin R*	CAATTACTCCAACCCGAATGGC
*Pax6 F*	GAGCCTCATCTGAATCTTCTCCG
*Pax6 R*	CGTCCATCTTTGCTTGGGAAATC
*p53 S*	TTGGATCCATGTTTTGCCAACTGGCC
*p53 AS*	TTGAATTCAGGCTCCCCTTTCTTGCG
*PTEN F*	GGACGAACTGGTGTAATGATATG
*PTEN R*	TCTACTGTTTTTGTGAAGTACAGC
*SDF-1S*	GGGGGAATTCCATGAACGCCAAGGTCGTGGTC
*SDF-1 AS*	GGGGTCTAGAGGGCATGGATGAATATAAGCTGC
*Sox2 S*	ACCGGCGGCAACCAGAAGAACAG
*Sox2 AS*	GCGCCGCGGCCGGTATTTAT
*TIMP1 F*	ACT GGA AGC CCT TTT CAG AGC
*TIMP1 R*	AAT TCC GAC CTC GTC ATC AGG
*VEGF AS*	CGATCGTTCTGTATCAGTCTTTCC
*VEGF S*	GAAGTGGTGAAGTTCATGGATGTC

Note: F, forward; R, reverse; S: sense; AS, anti-sense. All the primers were described previously [104].

### Immunocytochemistry

The immunocytochemistry used has also been previously described [105]. Cells were grown on Matrigel-coated chamber slides and selective antibodies were applied after fixation and permeabilization. Images were taken on a Zeiss LSM 510 Meta Microscopy System using 40x or 63x objectives or an Olympus IX-70 fluorescence microscope using 4x, 10x, 20x, 40x, or 100x objectives.

### Western blot analysis

The Western blot analysis used has also been previously described by us [83,106,107]. Briefly, cells cultured in one 10-cm dish were washed three times with PBS, collected, and incubated in 500 μl of lysis buffer (10 mM Tris, pH 7.5, 50 mM NaCl, 1 Triton X-100) for 30 min at 4°C. Lysates were clarified by centrifugation at 15,000x*g* for 15 min. After preclearing, supernatants were quantified with a protein assay. Fifty micrograms of the lysate protein were mixed with SDS-PAGE loading buffers and loaded into a lane, which was subjected to resolution by SDS-PAGE. The sample was subjected to immunoblot analysis with Caveolin-1 mouse monoclonal antibody (4 H312, sc-70516; Santa Cruz Biotech). Equivalent amounts of total cell lysates were loaded into all the lanes.

### Stereotactic surgical procedure with NOD/SCID mice

All animal protocols were approved by our IACUC. Immune-deficient mice (NOD/SCID, 6–8 weeks old) were used. Animals were anesthetized with an intraperitoneal injection of a Ketamine/Xylazine cocktail (132 mg/kg Ketamine + 8.8 mg/kg Xylazine), were immobilized in a stereotactic apparatus and received stereo tactically-guided injections of CD133+ cells into the right frontal lobe (~2 mm lateral and 1 mm anterior to bregma, at a 2.5 mm depth from the dural surface). The glioma cell line U87 (from ATCC, Manassas, VA) was used as a control. Injections were performed through a burr hole drilled into the skull after a skin incision. 6x10^3^-6x10^4^ of cells in 2 ul of PBS were injected with a 30 gauge 5 ul Hamilton syringe over a 3–5 minute period. After retracting the needle over a 2–4 minute period, bone-wax was used to occlude the burr hole, betadine applied to surgical area, and the skin was closed with skin glue or sutures. Post-surgical mice were kept on a heating pad to recover and eye ointment was applied.

### Histological analysis of mouse brain

Prefixation was performed by transcardiac perfusion with lactated Ringer’s solution followed by 4 buffered-paraformaldehyde. The brains were postfixed and embedded with paraffin and cut with a microtome. Brain sections were mounted on slides and stained with Harris’ hematoxylin then counterstained with alcoholic eosin.

## Abbreviations

CT: Computed tomography; CSCs: Cancer stem cells; GBM: Glioblastoma multiforme; MRI: Magnetic resonance imaging; NSCs: Neural stem cells.

## Competing interests

The authors declare that they have no competing interests.

## Authors’ contributions

SCL conceived of the study, designed with coordination, carried out tumor processing and CSC isolation and in vitro and ex vivo culture, and drafted the manuscript. LTV carried out the PCR and Western blotting studies. HWH and WGL performed the surgery and analyzed MRI images. VK carried out the immunocytochemistry with technical help from AS. ZM performed all pathological analyses. QL, JW, and HZ carried out in vivo studies. HZY and JHW helped perform ex vivo studies. PHS participated in neural stem cell culture and advised on editing of the manuscript. WGL advised on conceiving of the study, participated in its design and coordination, and helped draft the manuscript. All authors read, revised, and approved the final manuscript.
